# *In silico* design and screening of hypothetical MOF-74 analogs and their experimental synthesis†
†Electronic supplementary information (ESI) available: S1 Benchmarking simulated CO_2_ adsorption; S2 synthesis of Mg_2_(olsalazine); S3 additional characterization of Mg_2_(olsalazine); S4 MIL-53 constraints example; S5 Crystallographic Information Files (CIFs) for MOF-74 analog library; S6 experimental adsorption studies; S7 a sample CP2K input file. See DOI: 10.1039/c6sc01477a


**DOI:** 10.1039/c6sc01477a

**Published:** 2016-06-21

**Authors:** Matthew Witman, Sanliang Ling, Samantha Anderson, Lianheng Tong, Kyriakos C. Stylianou, Ben Slater, Berend Smit, Maciej Haranczyk

**Affiliations:** a Department of Chemical and Biomolecular Engineering , University of California , Berkeley 94720 , USA; b Department of Chemistry , University College London , 20 Gordon Street , London WC1H 0AJ , UK; c Laboratory of Molecular Simulation , Institut des Sciences et Ingénierie Chimiques , Ecole Polytechnique Fédérale de Lausanne (EPFL) , Rue de l'Industrie 17 , CH-1951 Sion , Valais , Switzerland; d Department of Physics , King's College London , The Strand , London , WC2R 2LS , UK; e Computational Research Division , Lawrence Berkeley National Laboratory , Berkeley , California 94720 , USA . Email: mharanczyk@lbl.gov; f IMDEA Materials Institute , C/Eric Kandel 2 , 28906 – Getafe , Madrid , Spain

## Abstract

In this work we present the *in silico* design of metal-organic frameworks (MOFs) exhibiting 1-dimensional rod topologies. We introduce an algorithm for construction of this family of MOF topologies, and illustrate its application for enumerating MOF-74-type analogs. Furthermore, we perform a broad search for new linkers that satisfy the topological requirements of MOF-74 and consider the largest database of known chemical space for organic compounds, the PubChem database. Our *in silico* crystal assembly, when combined with dispersion-corrected density functional theory (DFT) calculations, is demonstrated to generate a hypothetical library of open-metal site containing MOF-74 analogs in the 1-D rod topology from which we can simulate the adsorption behavior of CO_2_. We finally conclude that these hypothetical structures have synthesizable potential through computational identification and experimental validation of a novel MOF-74 analog, Mg_2_(olsalazine).

## Abstract 2

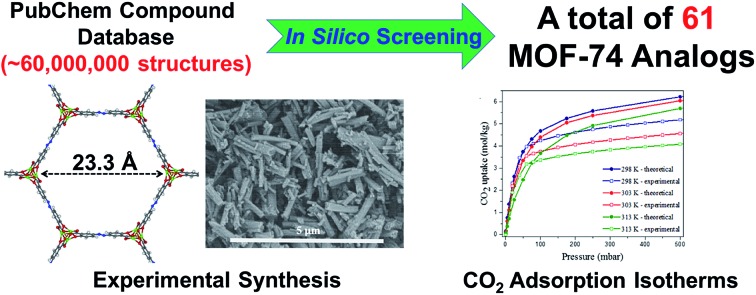
We present the *in silico design* of MOFs exhibiting 1-dimensional rod topologies by enumerating MOF-74-type analogs based on the PubChem Compounds database. We simulate the adsorption behavior of CO_2_ in the generated analogs and experimentally validate a novel MOF-74 analog, Mg_2_(olsalazine).

## Introduction

Metal–organic frameworks (MOFs) are a widely studied class of crystalline, nanoporous materials whose composition of metal or metal-oxide nodes connected by organic linkers results in highly tunable structures with diverse chemical and geometric properties.^1^ Thus these materials are poised to make a broad impact in a variety of technological applications such as carbon capture,^2^ natural gas^3^ and hydrogen storage,^4^ molecular sensing,^5^ catalysis,^6^ and other various separation processes.^7^ The discipline of reticular chemistry, or the rational design of MOFs from molecular building blocks in an underlying topology,^8^ has been utilized to design vastly diverse structures. Hence reticular chemistry has been a useful tool, both experimentally and computationally, to tune these materials' structural and chemical properties for a given application.

Previous computational work has been directed towards generating large databases of hypothetical MOFs *in silico* through a variety of methods. MOFs can be abstracted by a special type of graph known as a periodic net that contains the information of their underlying topologies, and resources such as the RCSR^9^ and EPINET^10^ databases have been created to store this information. Martin and Haranczyk used the connectivity information of molecular building blocks and these catalogued nets to assemble hypothetical materials *in silico* for various classes of nanoporous materials^11^ and a similar approach was later implemented by the Heine group.^12^ The connection-based assembly strategy of Wilmer *et al.*, which builds MOFs building block by building block, was shown to generate over 137 000 hypothetical MOFs with diverse geometric properties and linker functionalizations.^13^ Three hundred of these materials displayed a greater simulated methane storage capacity than any known material at the time. In addition to the assembly algorithm, a methodology for linker selection is also necessary for *in silico* design efforts. Previous methods to achieve this have relied on screening moderate-sized databases of commercially available molecules (∼7 000 000),^14^ using a genetic algorithm to investigate compositional space of possible linkers,^15^ or brute-force enumeration of linkers from fragments of known MOFs.^13^

These studies involving enumerated hypothetical databases have proven useful for, among other things, elucidating structure–function relationships. Yet a noticeable limitation is that most, with a notable exception of the MIL-47 and MIL-53 analogs of Wilmer, do not contain MOFs exhibiting a so called 1-D rod topology^16^ in which the metal–oxide secondary building units (SBUs) extend as a rod infinitely in one direction. MOFs exhibiting this topological feature have demonstrated extremely interesting behavior including remarkable CO_2_ capture in MOF-74^17^ and so called breathing frameworks^18^,^19^ which can yield stepped adsorption isotherms.

Yet thus far, reticular chemistry studies on 1-D rod MOFs have been almost exclusively limited to the experimental realm, with intense focus on exploring analogs of MOF-74 to increase its already remarkable potential for CO_2_ capture. One of the original works in this area performed an isoreticular expansion of MOF-74 through exchange of the 2,5-dioxidobenzene-1,4-dicarboxylate (DOBDC) linker for ligands extended by an increasing number of benzene rings,^20^ thereby increasing the channel volume of MOF-74. Other efforts have aimed to tune or alter the open-metal site chemistry by utilizing different metal elements during synthesis^21^,^22^ or through functionalization of the open-metal site by appendage of amine based molecules.^23^

Our reticular chemistry study combines an application that has only been studied experimentally, the creation of MOF-74 analogs, with a computational method for the automated generation of hypothetical analogs of MOFs exhibiting a 1-D rod topology. In place of brute-force enumeration of hundreds or thousands of structures across various topologies seen in previous *in silico* studies, we take a more directed approach to investigate the MOF-74 structure and vary the chemical composition of the ligand to alter its geometric and chemical properties. While this has been done previously for structures in the simpler MOF-5 topology,^14^ MOF-74 requires a novel building algorithm since it exhibits 1-D SBU rods and complex connectivity between ligands and SBUs. Furthermore, in this study we explore the chemical space represented by the ∼60 000 000 distinct chemical species in the PubChem Compounds database^24^ and exchange linkers found within this space with the DOBDC linker in the original structure. Only 61 molecules (0.0001% of this database) are identified as feasible MOF-74 analog linkers, which provides a concise starting point for attempts at experimental synthesis. One ligand in the set that was also commercially available, known by it’s pharmaceutical name olsalazine or 3,3′-azobis(6-hydroxybenzoate)salicylic acid, was used to successfully synthesize a MOF-74 analog. We significantly increase the impact of our *in silico* screening by demonstrating that novel, predicted structures are indeed synthesizable.

Hence we develop a hypothetical structure generation method using a geometric optimization routine which permits the rapid *in silico* assembly of 1-D rod MOF analogs (specifically MOF-74 structures in this study) while only using linkers guaranteed to represent an experimentally realizable molecule. The library of hypothetical MOF-74 analogs was structurally analyzed and screened for CO_2_ capture potential by comparison to the performance of the original structure based on the DOBDC linker. Finally, the knowledge ascertained from this computational study was utilized to direct efforts to synthesize a particular MOF-74 analog from a commercially available linker, yielding an experimental structure in excellent agreement with the *in silico* generated and optimized structure. Thus we have introduced a set of experimentally realizable structures that can now be targeted in future synthetic efforts.

## Methods

The overall workflow for *in silico* generation, analysis, and experimental synthesis of hypothetical MOF-74 analogs consists of five steps and is summarized below:

1. *Identification of potential ligands*. Chemical substructure searching and ligand conformational analysis is performed to select potential substitutes for the MOF-74 DOBDC linker from the PubChem Compounds database.

2. *Crystal structure assembly*. Potential ligands are inserted into the MOF-74 topology through geometric optimization of the crystal structure.

3. *Dispersion corrected DFT*. Dispersion corrected DFT calculations are performed to optimize each assembled structure. REPEAT^25^ derived partial atomic charges are obtained following the optimization, required for step 4.

4. *Grand Canonical Monte Carlo (GCMC) simulations*. CO_2_ adsorption is simulated at flue gas conditions with GCMC for each structure in the library of MOF-74 analogs.

5. *Prioritization of synthetic targets*. Hypothetical crystal structures constructed from commercially available linkers are identified as synthetic priority targets. The hypothetical MOF-74 analog, hereon named Mg_2_(olsalazine), was synthesized from the commercially available olsalazine linker to validate our approach of generating experimentally realizable crystal structures *in silico*.

**Fig. 1:**
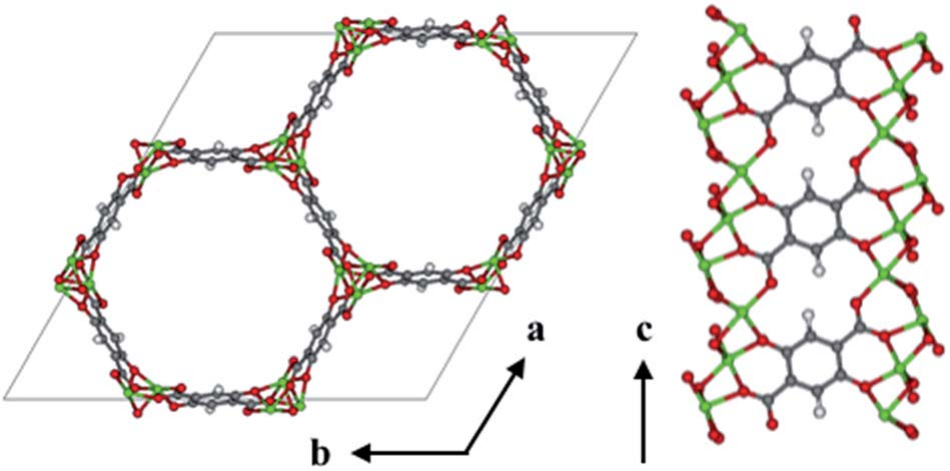
The *ab* face of Mg-MOF-74 illustrates the hexagonal channel shape. A slice of the channel wall demonstrates the extension of the 1-D metal–oxide (Mg–O, Green-Red, respectively) rods in the *c*-direction and the connectivity of the DOBDC linker to two adjacent rods.

The visualization of important structural features in MOF-74, shown in Fig. 1, is intended as a visual aid for the reader throughout the Methods section. This crystal exhibits infinite 1-D hexagonal channels, where adjacent metal-oxide rods are bridged by a DOBDC linker. A DOBDC linker connects to each rod through its two carboxylic acid oxygen atoms and an adjacent phenolic oxygen.

### Identification of potential ligands

**Fig. 2:**
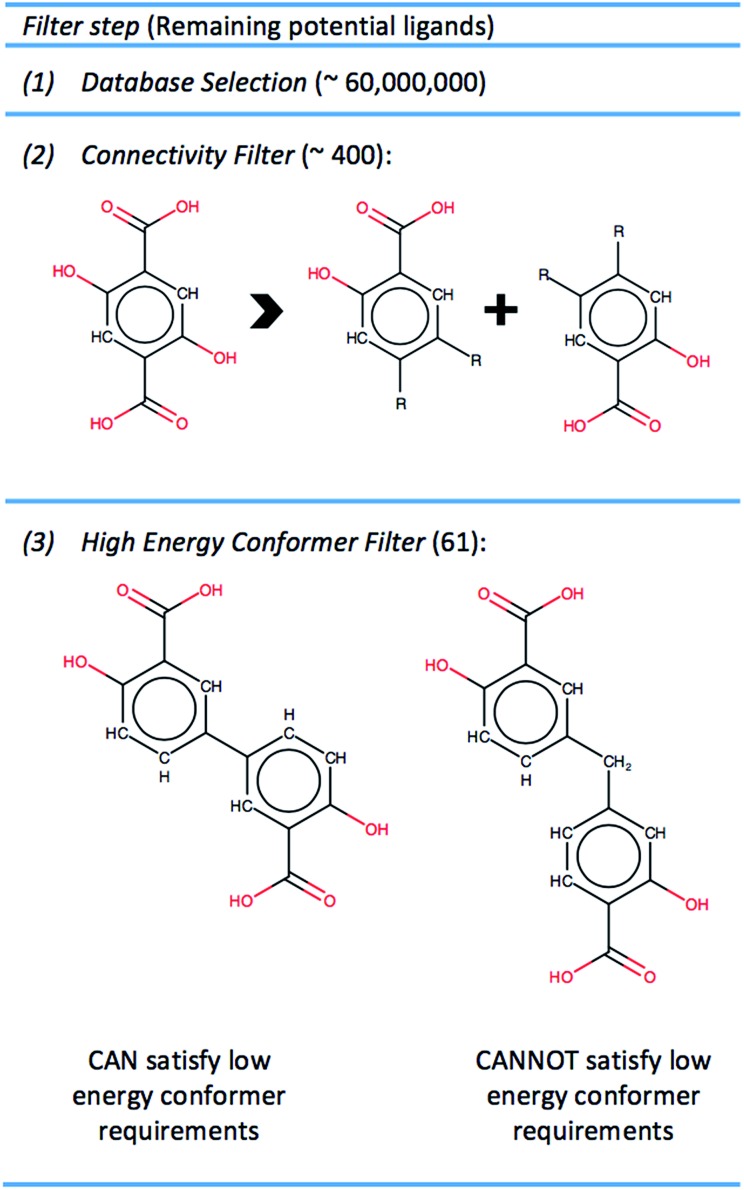
Step 1: PubChem Compounds database is chosen to screen ∼60 000 000 chemicals. Step 2: filtering out molecules not appropriate for MOF ligands and those that do not satisfy MOF-74 chemical connectivity removes all but 0.0007% of compounds. Step 3: molecules that cannot achieve a low energy conformer compatible with MOF-74 geometric requirements are filtered, thereby removing 84% of the remaining potential ligands.

To create a library of hypothetical MOF-74 analogs requires identifying linkers which can feasibly be exchanged with the DOBDC linker in the original structure. The steps through which ligands were identified and chosen for MOF-74 enumeration is summarized in Fig. 2.

#### (1) Database selection

For this study we expand the scope of our exploration of chemical space from a commercial database such as eMolecules (https://www.emolecules.com), sometimes referred to as the ‘; http://amazon.com’ for chemicals, to the larger PubChem Compounds database. This PubChem library of molecules consists of ∼160 000 000 user deposited structures, from which 60 000 000 are chemically validated and determined to be unique. This subset of unique molecules is known as the PubChem Compounds database and is approximately nine times larger than the eMolecules database. While these PubChem compounds may not necessarily be commercially available, any ligand identified from this library should indeed represent a feasible, experimentally realizable chemical structure.

#### (2) Connectivity filter

The chemical connectivity of MOF-74 demonstrates greater complexity than the connectivity seen in the broad class of MOFs where the ligand attaches to the SBUs by two or more carboxylic acid groups. Here we require a phenol group directly adjacent to the carboxylic acid group in order to have the correct connectivity. We impose the constraint that a molecule must have exactly two of these identical substructures shown in Fig. 2 to satisfy the particular chemical connectivity of MOF-74. This substructure can be abstracted as a SMARTs string, or a textual representation of a substructure within a molecular graph, which can be readily identified by the open-source software package OpenBabel.^26^ Thus each molecular entry in PubChem can be quickly analyzed and identified if it contains a particular SMART. After removing database entries that contained fragmented structures, salts, or net charged species and filtering for molecules that contained exactly two of the required SMARTs for MOF-74 connectivity, only ∼400 molecules remained as potential ligands. Thus simply identifying molecules with MOF-74 connectivity indicates we can only investigate 0.0007% of the chemical space contained in the PubChem database.

#### (3) High energy conformer filter

**Fig. 3:**
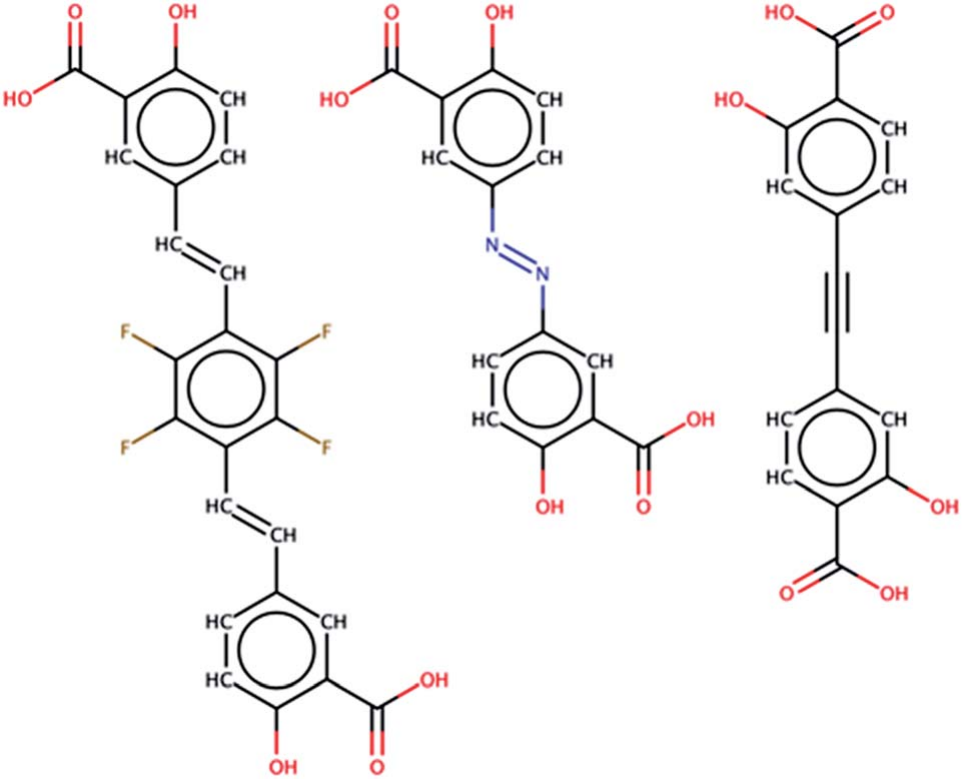
A sample of the 61 molecules found in the PubChem database that were identified as potential ligands for MOF-74 analogs. The middle linker, olsalazine, is utilized to synthesize a novel MOF-74 analog.

Additional filtering was required to identify potential linkers that could adopt a conformation suitable for building a MOF-74 analog. The DOBDC linker is connected to a given metal rod SBU by three Mg–O bonds, one at each carboxylic acid oxygen and a third at the phenolic oxygen (see Fig. 1). This third connection point from the linker to the SBU adds the constraint that the terminal benzene ring which connects to the SBU is not free to rotate about the axis defined by the carboxylic acid bond vector. Since the original linker consists of just one benzene ring, any proposed linker from which we attempt to build a MOF-74 analog must have terminal benzene rings that lie in the same plane. An additional requirement is imposed in which the angle between the two vectors represented by the carboxylic acid carbon to benzene ring bond in both connection groups are required to have an angle of 180 degrees or 120 degrees. If the angle between the vectors is 180 degrees, then the assembled crystal structure will be a variant of the originally published MOF-74 structure.^27^ If the angle between vectors is 120 degrees, then the assembled structure will be a variant of the *m*-MOF-74 structure.^21^ These orientation constraints significantly affect the number of linkers that can be used to enumerate MOF-74 analogs. The Confab tool in OpenBabel, which systematically generates diverse, low-energy conformers,^28^ was used to generate a set of low-energy conformers for each of the 400 remaining linkers. The criteria for a low-energy conformer was set at 50 kJ mol^–1^ above the minimum energy conformer in vacuum and was calculated *via* the Merck Molecular Force Field (MMFF94).^29^ The molecules that passed the conformational test were those that contained at least one conformer in which the planes defined by the terminal benzene atoms were within ±10% of 180 degrees and carboxyl acid bond vectors that were within ±10% of 120 degrees or 180 degrees. Discarding molecules that could not realistically achieve the required conformational constraints of MOF-74 resulted in the removal of all but 61 structures from the set of potential ligands, yielding a collection of molecules representing 0.0001% of the PubChem database. While not a quantitative predictor of the synthesizability of a MOF-74 analog, the use of only low energy conformers suggests that the assembled MOF-74 analog may be experimentally realizable. Three of the 61 selected linkers in the PubChem database are shown in Fig. 3. Each ligand has access to a low energy conformer that exhibits both planarity between the terminal benzene rings and an angle between the two carboxylic acid C–C bonds of either 120 degrees or 180 degrees. All remaining linkers can be seen in the assembled MOF-74 analogs by visualizing the CIFs given in S5 of the ESI.†

### Crystal structure assembly

**Fig. 4:**
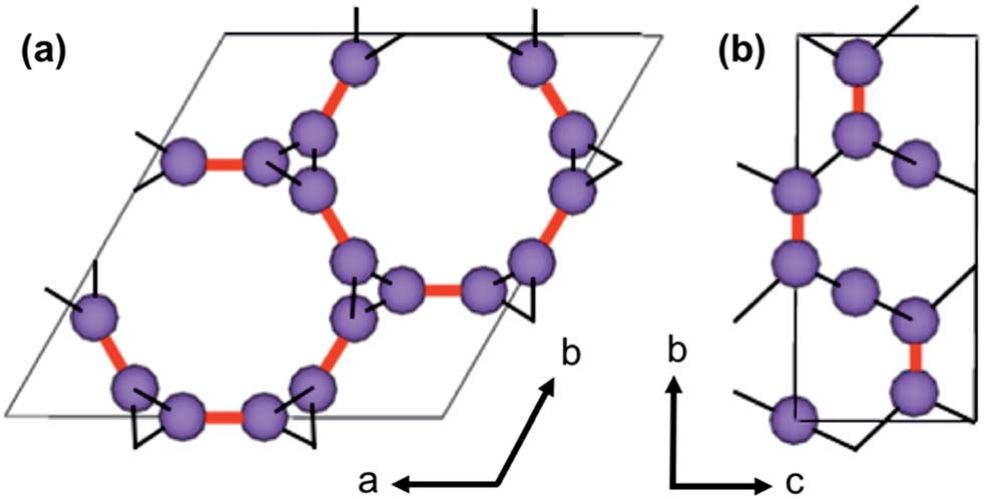
The **etb** net representation of MOF-74 where purple spheres represent nodes and lines represent edges in the graph. Nodes correspond to the metal atoms in each rod, red lines are edges that abstract a ligand in MOF-74, and thin black lines abstract connections within a single SBU rod. (a) View of the *ab* face of **etb** net. (b) View of the *bc* face of the **etb** net.

The **etb** net of MOF-74, visualized in Fig. 4, exhibits special complexities which preclude the use of previously developed methods for *in silico* crystal design.^11^,^13^ Three distinct problems arise. Firstly, not all edges in nets that abstract a 1-D rod MOF represent a linker or segment of a linker. As seen in Fig. 4, only some edges (highlighted in red) in the **etb** net abstract the ligand that connects one SBU rod to another, while others abstract edges that exist within the SBU rod itself (highlighted by thin black lines). Secondly, each node in **etb** does not abstract a single, discrete SBU in MOF-74 but rather they abstract only the metal atom contained within the SBU rod. For example, all nodes that can be visited without traversing a red edge represent the three metals contained by one rod within a single unit cell. Thirdly, attempting to define the *c*-component of the SBU rod's central (*a*, *b*, *c*) coordinates becomes ambiguous since the rod transverses periodic boundary conditions in the *c*-direction. Thus the process of embedding the SBU rods in three dimensional space can significantly change depending on the selected linker. Upon exchange of the red edges in Fig. 4b with an alternative linker to DOBDC, the edge may in fact no longer be parallel to the *ab* face and all the SBU rods will shift relative to each other in the *c*-direction based on the new ligand's geometry. These three difficulties necessitate a new methodology to quickly assemble crystal structures from the 61 selected ligands.

**Fig. 5:**
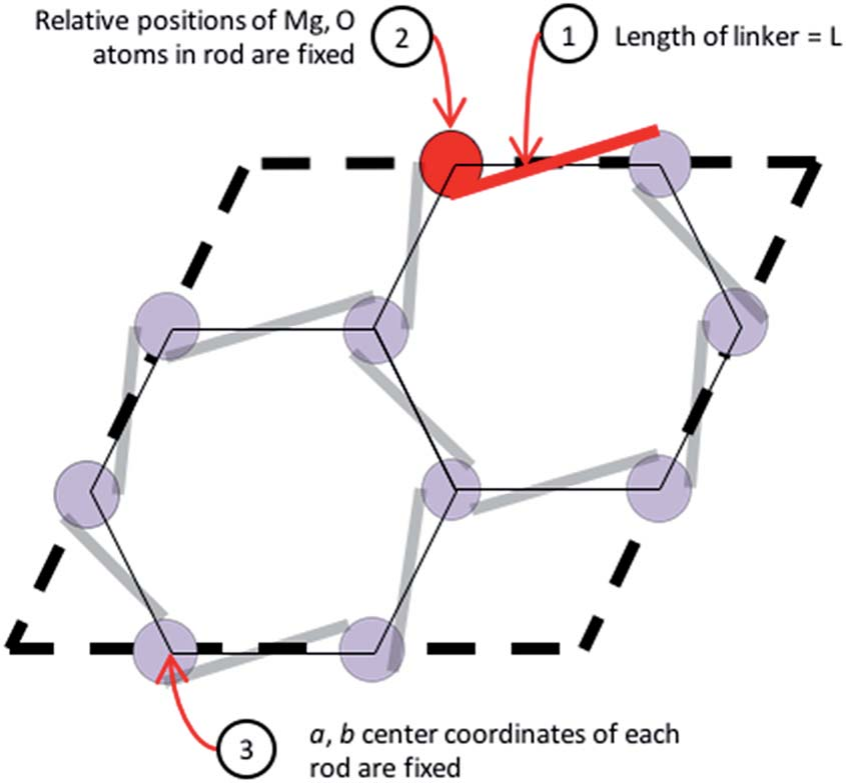
A simplified geometric representation of the MOF-74 unit cell. Three constraints dictate how the SBU rods are able to position themselves relative to one another in the crystal framework.

We overcome these challenges by simplifying our view of the MOF-74 net topology and by using a simple geometric optimization routine to assemble a new crystal structure for a given ligand. We identify three geometric constraints that must remain invariant after ligand exchange, schematically represented in Fig. 5. Note that these constraints are net independent and could therefore be identified for any MOF whose metal oxide SBUs are indeed 1 dimensional (an example of MIL-53 is shown in S4 of the ESI†). Firstly, the length of a ligand, L, is quantified as the distance between the two carboxylic acid carbons since they represent atoms that are immediately connected to the SBU rods. Thus they are termed the connection points of each rod. Upon enumerating a new crystal structure, we require that the magnitude of the distance between the connection points on two adjacent rods be equal to the length of the ligand. Secondly, the chemical environment within the SBU rod is invariant, so the relative coordinates of all Mg and O atoms in the rod are fixed. Thirdly, the *a* and *b* fractional coordinates of the center of all rods must remain constant to preserve the hexagonal packing of the crystal structure.

**Fig. 6:**
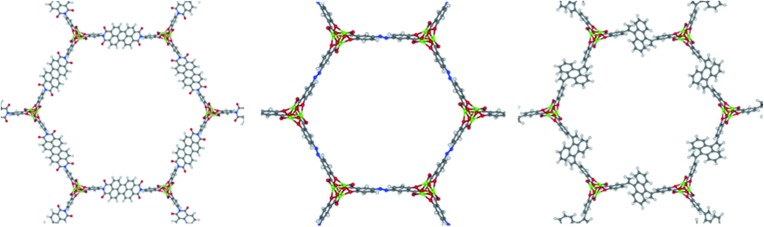
Visualization of three of the constructed hypothetical MOF-74 analogs. The center structure is Mg_2_(olsalazine).

By imposing these constraints, we can establish the framework of the unit cell by resolving the only two variables that can change for any given ligand. Firstly, an SBU rod may shift in the *c*-direction relative to its starting *c*-coordinates. Secondly, the unit cell will expand by some fractional value in both the *a* and *b* crystallographic directions. These variables we denote dC and *F*, respectively. The *c* lattice parameter cannot increase or decrease upon ligand exchange due to the connectivity of a 1-D rod MOF. Thus, with L as an input, we can solve for the values of *F* and dC that satisfy the three constraints of Fig. 5. A continuum of (*F*, dC) solutions exist; if *F* increases for example, dC must also change in order to maintain a fixed length between connection points on adjacent rods. The final task is to choose an (*F*, dC) from the set of solutions that yields the most feasible crystal structure. This is accomplished by implementing a 3D-point cloud fitting of all carboxylic acid and phenolic oxygen atoms on the terminal benzene rings to their known connection coordinates on an SBU rod. The (*F*, dC) combination that results in a point cloud fitting with the lowest root mean square displacement (RMSD) is selected as the optimal crystal framework. Thus the structural assembly method is fast and efficient, taking only a few seconds to generate all 61 hypothetical MOFs, three of which are visualized in Fig. 6.

### Geometry relaxations and partial atomic charge analysis

The geometries, including both cell parameters and atomic positions, of the 61 hypothetical MOF-74 analogs generated from the aforementioned procedure are optimized at DFT level of theory, using the PBE functional,^30^ together with Grimme's D3 dispersion correction^31^ with the Axilrod–Teller–Muto three-body terms. The optimized lattice parameters of the original Mg-MOF-74, *a* = 26.17 Å and *c* = 6.95 Å, are in good agreement with experimental values (*a* = 25.92 and *c* = 6.86).^32^ Similar settings were used in our previous studies on MIL-53 and UiO-66 type MOFs,^33^–^35^ in which good agreement with experimental structural and calorimetric data was obtained. All periodic DFT calculations were performed using the CP2K code which uses a mixed Gaussian/plane-wave basis set.^36^,^37^ We employed double-*ζ* polarization quality Gaussian basis sets and a 400 Ry plane-wave cutoff for the auxiliary grid, in conjunction with the Goedecker–Teter–Hutter pseudopotentials.^38^,^39^ A convergence threshold of 1.0 × 10^–6^ Hartree was used for all self-consistent field calculations. The structural optimizations were considered converged if the maximum force on all atoms falls below 0.534 kcal mol^–1^ Å^–1^ (4.5 × 10^–4^ hartree per bohr). All calculations were performed with the *Γ*-point approximation using a 1 × 1 × 2 multiplication of the hexagonal primitive cell.

The partial atomic charge analysis was performed at the relaxed geometry, using the REPEAT method of Campañá *et al.*,^25^ which was recently implemented into the CP2K code based on a restrained electrostatic potential framework.^40^ Our implementation of the REPEAT method in the CP2K code makes it possible to perform seamless geometry optimization and accurate partial atomic charge analysis of porous materials in the same software package. In addition, the parallel algorithms significantly accelerate the partial atomic charge analysis of large systems, for which the computational cost scales cubicly with the cell length and density of grid points used for the electrostatic potential fitting. The REPEAT method calculates partial atomic charges from electrostatic potentials determined from DFT calculations, and only the grid points outside the van der Waals (vdW) radii of each atom were included in the fitting. In our calculations, we have used the vdW radii from the Universal Force Field (UFF),^41^ which were also used by Campañá *et al.* in their original paper on the REPEAT method,^25^ with a scaling factor of 1.0. No other restraint was imposed during the REPEAT charge analysis. Our implementation of the REPEAT method is available in the latest released version of the CP2K code. A detailed comparison of the derived charges from our implementation and from previous calculations, and a sample input to perform REPEAT charge analysis with CP2K, are provided in the ESI.†

### Porosity characterizations and GCMC simulations

Geometric structure analysis of the MOF-74 analog set was completed using the Zeo++ software suite.^42^ A probe radius of 1.65 Å which corresponds to the kinetic diameter of CO_2_ was utilized to characterize the probe accessible surface area, the probe accessible volume, the largest included sphere, and the largest free sphere of all hypothetical structures. GCMC simulations at flue gas temperature of 313 K were utilized to measure the CO_2_ adsorption capabilities of the MOF-74 analog library. Force field parameters were obtained from previous studies^43^ in which DFT single-point energy calculations were performed along a guest molecule's approaching path of minimal repulsion to each unique atom type in MOF-74. The energies were parameterized with the Buckingham potential to obtain highly accurate pairwise interaction parameters that are able to describe the repulsive interaction of CO_2_ with the excess electron density at an open metal site, an essential requirement for capturing the adsorption behavior of CO_2_ in MOF-74. These pairwise parameters are available for the atom types present in the original MOF-74 structure; however, all MOF-74 analogs contain at least one unique atom type in the crystal that is not present in the original structure because all 61 ligands have more atom types than the original DOBDC linker. UFF was utilized to describe non-bonded, pairwise interactions between CO_2_ and these atom types not found in the original MOF-74 structure.

### Prioritization of synthetic targets

The olsalazine molecule was identified as one of the few commercially available ligands in the set of hypothetical MOF-74 analogs that have not been synthesized yet and was therefore targeted for experimental synthesis. The synthetic procedure is outlined in S2 of the ESI.†

## Results and discussion

### Quality of structural assembly

**Fig. 7:**
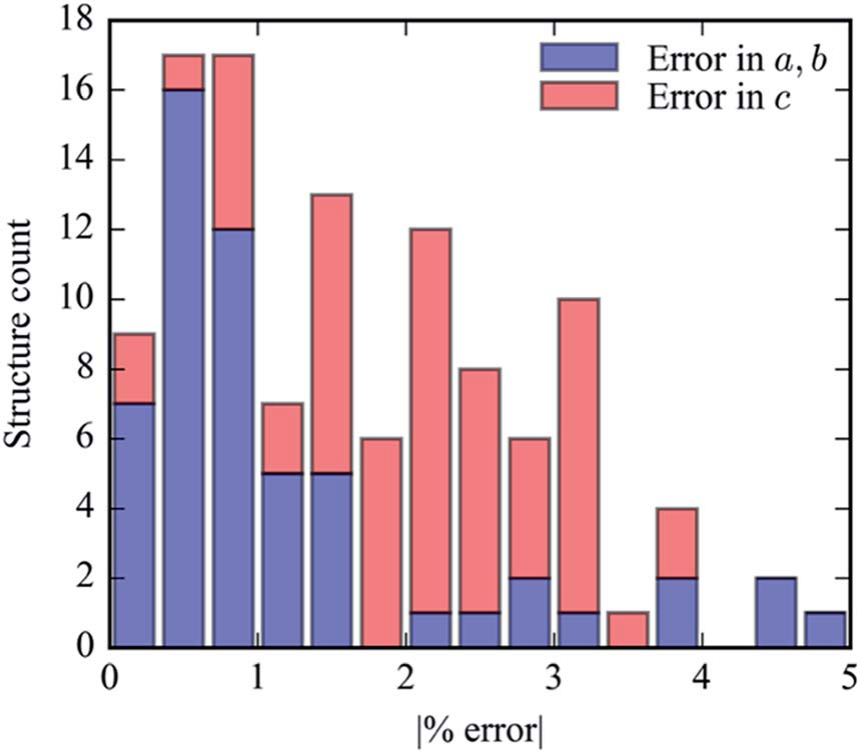
The absolute % error in the magnitudes of each lattice cell parameter from pre-DFT relaxed structures. DFT optimization results in very little change in the lattice cell parameters for all structures.

Our geometric optimization routine was executed for each of the 61 potential linkers found within the PubChem Compounds database to generate a new hypothetical MOF-74 structure. The accuracy of this methodology in generating reasonable lattice geometries can be evaluated by observing the unit cell parameters both before and after DFT relaxation. Fig. 7 illustrates how DFT relaxation very marginally alters the lattice cell parameters. The accuracy of the generated crystal structures can be attributed to our prescreening of linkers to find low energy conformers. Since the new *a* (and *b*) lattice cell parameters are dependent on the length of the proposed linker, using an energy minimized linker conformation results in a crystal structure that changes minimally following DFT relaxation. The error in the *c* parameter tends to be slightly larger than the error in the *a* parameter since this value is constant in the crystal assembly algorithm and cannot be pre-optimized by ligand conformational analysis.

### Geometric analysis of hypothetical structures

**Fig. 8:**
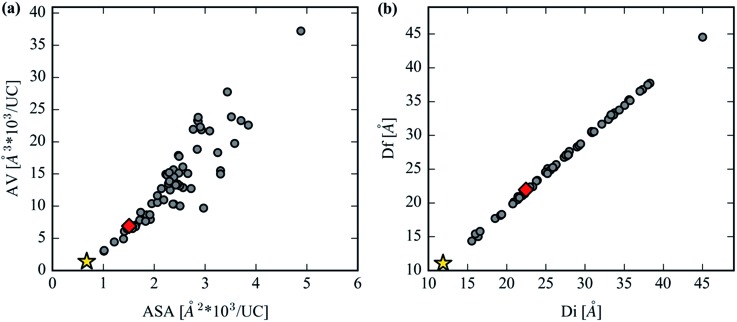
(a) The volume accessible to a 3.3 Å diameter probe per unit cell *versus* the accessible surface area per unit cell. (b) The free sphere, *D*_f_, plotted against the largest included sphere, *D*_i_, for the hypothetical MOF-74 library. The gold star represents Mg_2_(DOBDC) and the red diamond represents Mg_2_(olsalazine).

An interesting comparison can be drawn between the CO_2_ accessible volume (AV) and the CO_2_ accessible surface area (ASA), shown in Fig. 8a. The unconventional choice of ASA and AV units were made so that comparisons between structures' geometric properties are made on an absolute basis (Å^3^ per UC or Å^2^ per UC) that does not account for the unit cell volume or crystal density, two quantities which vary widely across the analog library. After the AV of the unit cell reaches a critical volume of around 10 000 Å^3^, linkers are able to produce variations in ASA because the length of the inserted linker permits variations in molecular structure, such as functional side groups or ring twisting in the interior of the linker, that still satisfy the constrained linker environment of the MOF-74 topology. For example, structures in the 10 000 Å^3^ range have a 50% variation in their range of ASA, whereas at AVs below 10 000 Å^3^ there is a much narrower, almost negligible distribution in ASA. This presents unique opportunities to make 1-D apertures that are accessible to extremely large guest molecules whose channel walls are much rougher than the original MOF-74 structure. These variations in channel wall shape, combined with the extremely large free spheres shown in Fig. 8b, suggest that the capture and separation of larger molecules could be a useful application for these analog structures. MOFs with such a large aperture have been previously synthesized,^20^ and indeed one analog in our set, the novel Mg_2_(olsalazine), was synthesized and characterized in this study. The validation of this structure is described later on. Due to the close packing of linkers in the crystallographic *c*-direction, all analogs strictly maintain channels with a dimensionality of one since the largest included sphere and largest free spheres are almost exactly equivalent for all structures.

### CO_2_ capture in MOF-74 analogs

**Fig. 9:**
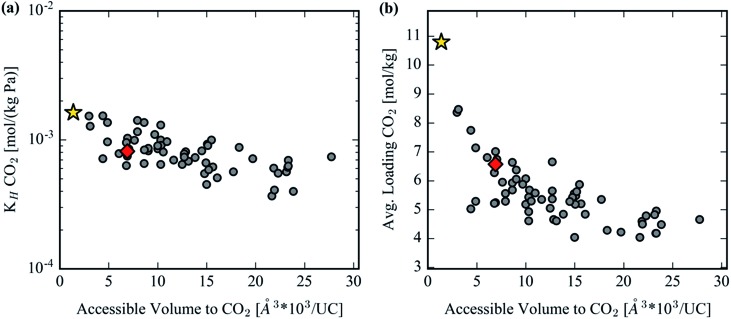
(a) CO_2_ Henry coefficient in the hypothetical MOF-74 library. (b) Average absolute CO_2_ loading in MOF-74 analogs at 313 K and 1 bar. The gold star represents Mg_2_(DOBDC) and the red diamond represents Mg_2_(olsalazine).

**Fig. 10:**
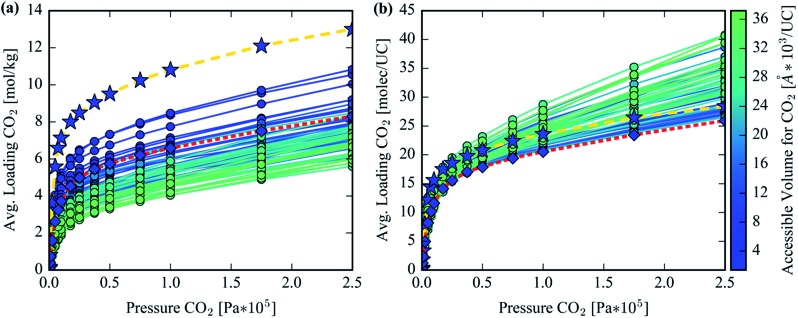
(a) CO_2_ adsorption isotherms in units of moles loaded per kilogram and (b) in units of molecules loaded per unit cell in the hypothetical MOF-74 analog library. Each isotherm is color-coded by the CO_2_ accessible volume per unit cell of the corresponding structure. The isotherm with star markers and gold dashes represents the isotherm for Mg_2_(DOBDC), whereas the isotherm with diamond markers and red dashes represents Mg_2_(olsalazine).

GCMC simulations were utilized to study the adsorption of CO_2_ in the hypothetical set of analogs to gauge their potential to reduce the parasitic energy^44^ of carbon capture for the original MOF-74 structure. Benchmarking with previous results and experimental data is provided in S1 of the ESI.† The Henry coefficient *versus* AV plotted in Fig. 9a shows a declining trend but with significant scatter. Many of the structures achieve comparable Henry coefficients to the original structure even with a 6-fold increase in the AV. This is not surprising as the Henry coefficient should not suffer significantly from simply increasing the pore size, yet it confirms that many of the analogs can achieve the remarkable CO_2_ uptake of the original MOF-74 in the limit of infinite dilution. At higher pore volumes the uptake of CO_2_ drops significantly, however. The spatial dilution of the open-metal sites by inserting extended ligands into the framework is not compensated by the addition of any binding sites as strong as the under coordinated metals. Fig. 9b clearly demonstrates the decrease in performance in CO_2_ loading after exchanging ligands to make a hypothetical MOF-74 structure. Furthermore, visualization of the CO_2_ isotherms, color-coded by each structure's accessible volume in Fig. 10, yields instructive insight. Fig. 10b reveals that, at the highest pressures, the structures with the largest channel volumes load more molecules per unit cell due to the increased free volume of each channel. All isotherms show a positive linear slope in the pressure regime of ∼2.5 bar indicating that CO_2_ is simply being compressed in the additional free space of these larger channels. The uptake per crystal weight demonstrates the opposite trend between loading and accessible volume. Fig. 10a clearly demonstrates the superiority of the original MOF-74 structure for CO_2_ loading capacity on a per crystal weight basis since the open metal sites are used most efficiently with the least amount of dilution by organic material.

We also note that, although we use a fully flexible model to generate ligand conformations for our optimized structural assembly, our GCMC simulations are performed with rigid host structures. In the experimental system extended linkers may induce flexibility in the framework. However, due to the connectivity of MOF-74 analogs' terminal benzene rings with the SBU rod *via* the two carboxylic acid oxygen atoms and phenolic oxygen atom, this terminal benzene is not free to rotate as mentioned in the previous section (3) High energy conformer filter. Therefore, the chemical and geometric environment directly surrounding the open metal sites is invariant even if the overall ligand exhibits flexibility. While the total pore volume may depend on flexibility, this will only affect loading behavior in the saturation regime of these extended frameworks. This high-pressure, saturation regime was not investigated in this study, as can be seen in Fig. 10, since it is less interesting for carbon capture applications and hence we expect flexibility to have a minimal effect on the results presented in this work.

### Validation of Mg_2_(olsalazine)

**Fig. 11:**
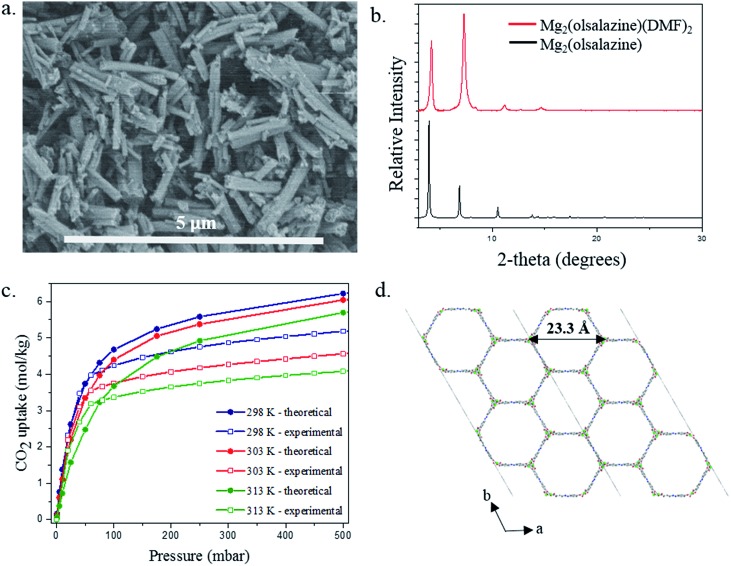
(a) SEM image of the as-made material reveals that the morphology of [Mg_2_(olsalazine) (DMF)_2_]·2DMF·3H_2_O crystals is based upon rods (scale bar: 5 μm) (b) comparison the simulated pattern from the idealized structure of [Mg_2_(olsalazine)] with the experimental pattern of the as made [Mg_2_(olsalazine) (DMF)_2_]·2DMF·3H_2_O (c) comparison of the theoretical and the experimental CO_2_ adsorption isotherms collected at 298, 303 and 313 K and 1 bar and (d) view of Mg_2_(olsalazine) along *c*-axis showing the formation of a 3-dimensional framework with hexagonal one-dimensional pores as it is observed in Mg-MOF-74.

The reaction of olsalazine sodium with Mg(NO_3_)_2_·6H_2_O in a mixture of DMF:ethanol (ratio of 1 : 1) at 120 °C yields a homogeneous material based on rod-type crystals as confirmed by scanning electron microscopy (SEM) images (Fig. 11a). The size of the isolated crystals was too small to permit structural resolution *via* single crystal X-ray diffraction. Despite this, powder X-ray diffraction confirms that the material is highly crystalline and the simulated pattern from the idealized structure of [Mg_2_(olsalazine)] shows an excellent agreement with the experimental pattern of the as-made [Mg_2_(olsalazine) (DMF)_2_]·2DMF·3H_2_O (Fig. 11b). In addition, the IR spectra of sodium olsalazine and [Mg_2_(olsalazine) (DMF)_2_]·2DMF·3H_2_O show that the peak corresponding to the carbonyl C=O bond of the coordinated ligand and DMF is between 1559 and 1651 cm^–1^, confirming the formation of the MOF-74 analogue (Fig. S2†). Thus, [Mg_2_(olsalazine) (DMF)_2_]·2DMF·3H_2_O is a 3D framework, isostructural to Mg-MOF-74 and Mg_2_(DOBPDC) (DOBPDC = 4,4′-dioxidobiphenyl-3,3′-dicarboxylate) and is based on infinite chains of octahedral MgO_6_ units running along the *c*-axis, where each Mg(ii) is bound to five O atoms from the olsalazine ligand and one O atom from DMF. Mg_2_(olsalazine) consists of hexagonal one-dimensional channels with dimensions of 23.3 Å (Mg–Mg distance, including van der Waals radii) which are larger than the channels resulted in Mg_2_(DOBPDC) (18.4 Å) (Fig. 11d). TGA analysis shows that the guest molecules (DMF and H_2_O) and the coordinated DMF molecules are removed in the temperature range of 30–370 °C. The guest and coordinated molecules comprise 48.9% of the total weight of bulk [Mg_2_(olsalazine) (DMF)_2_]·2DMF·3H_2_O (resulting from elemental microanalysis), which is in agreement with TGA data (51.4%). Decomposition of the framework to form MgO starts at 500 °C and occurs in a single step (Fig. S3†). Interestingly, similar to MOF-74, this material can also be synthesized with different metals.‡
‡The group of Long (UC Berkeley) has synthesized a metal analog series of this material as well (private communication).


CO_2_ adsorption isotherms were experimentally measured (*via* the procedure outlined in S6 of the ESI†) to further characterize the material and to directly test our theoretical predictions of the CO_2_ adsorption behavior. The theoretical and experimental isotherms are in good agreement, especially at low pressure. At higher pressures the experimental isotherms demonstrate that the material has lower saturation loading than the theory predicts. The GCMC simulations are performed assuming a perfect, fully accessible crystal structure. From an experimental point of view, it is difficult to obtain a perfect crystal; defects that cause pockets of inaccessibility to CO_2_ in the Mg_2_(olsalazine) powder would give a lower saturation loading in the experimental material. In fact, the experimental BET surface area was calculated to be 2331 m^2^ g^–1^ (see S6 of the ESI†), while the theoretical accessible surface area for N_2_ was computed to be 2763 m^2^ g^–1^ with Zeo++, thereby suggesting that indeed we do not have 100% crystal accessibility in the Mg_2_(olsalazine) powder. Ultimately, these experimental results demonstrate the theoretical methods used (*in silico* crystal design, DFT geometry optimization, *ab initio* partial atomic charge analysis, force field parameterization, and GCMC simulations) not only correctly predict the crystal structure but also its CO_2_ adsorption behavior.

## Conclusions

We have outlined a new, systematic method for enumerating MOFs exhibiting a 1-D rod topology. This method could be generally extended to systems other than the **etb** net and MOF-74. The geometric optimization approach permits the rapid and automated enumeration of analog structures, and our focus on searching for linkers in the PubChem database limited the structural assembly process to MOFs whose ligands are experimentally validated molecular species. The paucity of linkers found within PubChem (0.0001% of all molecules) that satisfy the strict geometric constraints of the MOF-74 topology provides a concise starting point for experimental efforts to synthesize analog structures. By providing an *in silico* methodology for generating 1-D rod MOFs as complex as MOF-74, we expand upon our ability to generate and predict properties of potentially interesting crystalline materials. Furthermore, we demonstrate the experimental validity of our *in silico* generated library by targeting the olsalazine linker for MOF synthesis. Mg_2_(olsalazine) was synthesized and its structural characterization and CO_2_ adsorption behavior were in good agreement with the *a priori* theoretical predictions.

Geometric analysis of the constructed hypothetical MOFs indicates that a wide range of surface areas are possible for a given accessible volume. This roughness and functionality of the channel walls, combined with the extremely large channel volumes, suggests that these MOFs could have greater potential for separations applications involving larger molecules than CO_2_. Physical and chemical variations in the channel surface would provide regions of preferentially favorable interactions for adsorption of various large molecules, whereas for CO_2_ capture, any new ligand in the analog library simply dilutes the open metal site density and does not provide any new, equally strong binding sites. Ultimately we have introduced 61 hypothetical MOF-74 analogs and demonstrated their potential synthesizability. Future experimental and computational efforts are two-fold: synthesize additional hypothetical structures and investigate their potential for separation processes of large molecules.

## Supplementary Material

Supplementary information

Supplementary information

Supplementary information
