# Supplementary material for: In silico design and screening of hypothetical MOF-74 analogs and their experimental synthesis
Source: Chem Sci. 2016 Jun 21;7(9):6263–72. doi: 10.1039/c6sc01477a (PMC6024208; doi:10.1039/c6sc01477a)
Supplement: Supplementary file 2 [file SC-007-C6SC01477A-s002.pdf]

Electronic Supplementary Material (ESI) for Chemical Science.
This journal is © The Royal Society of Chemistry 2016

Electronic Supporting Information (ESI)

S1. Benchmarking Simulated CO_2 Adsorption with Previous Results

A comparison of the partial charges between previously published work (VASP/REPEAT)[1] and this work (CP2K/REPEAT) are shown in Table S1 for the original MOF-74 structure.

Table S1. Summary of partial atomic charges derived from VASP (previous work) and CP2K (this work). The naming convention of atom types can be found in the previous work.[1]

Atom type	VASP/REPEAT	CP2K/REPEAT
Mg	1.56	1.66
Oa	-0.90	-0.92
Ob	-0.75	-0.76
Oc	-0.90	-0.97
Ca	0.90	0.88
Cb	-0.31	-0.32
Cc	0.46	0.48
Cd	-0.23	-0.26
H	0.19	0.21

The electrostatic potential generated by CP2K results in REPEAT derived charges that are 6% (Mg atom) and 8% (Oa atom) different than previous work. This difference only results in a small over-prediction of the CO_2 loading at low pressures on the CO_2 adsorption isotherm as seen in Figure S1, thereby validating the approach we have taken to relax structures and obtain partial charges. We note small differences in the derived REPEAT charges exist. This is because the electrostatic potentials are sensitive to computational settings, and different codes employ different basis sets and pseudopotentials. In particular, for the implementation of the REPEAT method in CP2K, in order to use the same integration method and grid as those used for the calculation of the DFT electrostatic potential, Gaussian charge densities with a fixed width are used in place of point charges. Nevertheless, we do not expect these technical differences will result in qualitatively different pictures on the calculated adsorption isotherms.

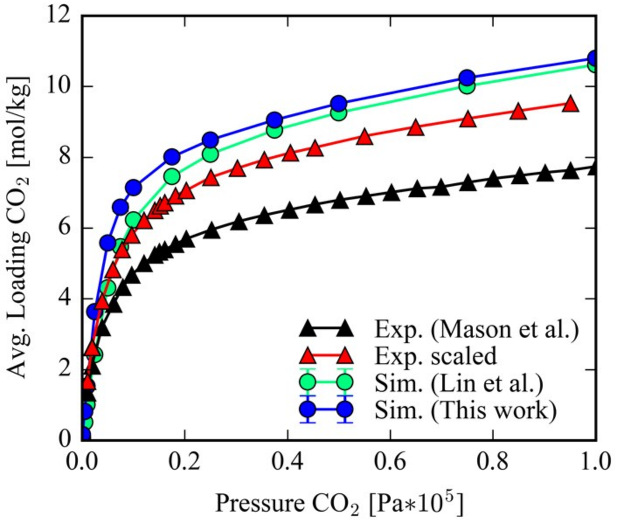

Figure S1. The CP2K relaxed structure and REPEAT derived atomic partial charges were validated by comparison of the CO_2 adsorption isotherm in the original MOF-74 structure with the previously published results of Lin et al. The experimental isotherm of Mason et al.[2] is scaled due to 80% accessibility of the material.

S2. Synthesis of Mg_2(olsalazine)

Materials. $Mg(NO_3)_2·6(H_2O)$, dimethylformamide (DMF) and ethanol were purchased from Sigma Aldrich whereas olsalazine sodium was obtained from AK Scientific. All reagents were used as received without further purification.

Synthesis of [Mg_2(olsalazine)(DMF)2]·2DMF·3H_2O. $Mg(NO_3)_2·6(H_2O)$ (25.6 mg, 0.1 mmol) and olsalazine sodium (13.8 mg, 0.04 mmol) were dissolved in a mixture of dimethylformamide (DMF) and ethanol (V_t: 3 mL, ratio 1:1) using 12 mL scintillation vials. The yellow solution was stirred for 10 min at room temperature and then it was heated at 120 °C for 72 hours with a heating rate of 0.2 ^oC/min. The reaction was gradually cooled to room temperature at a rate of 0.1 ^oC/min. Yellow rod type crystals of [Mg_2(olsalazine)$(DMF)_2$]·guest molecules were obtained with a yield of 36 % (based on $Mg(NO_3)_2·6(H_2O)$). Anal. Calcd for ([$C_{20}H_{20}N_4O_8Mg_2$]·2DMF·3H_2O): C 45.05, H 5.82, N 12.12. Found: C 44.21, H 5.71, N 12.28.

Phase purity of [Mg_2(olsalazine)$(DMF)_2$]·2DMF·3H_2O was demonstrated by powder X-ray diffraction (PXRD) data collected on a Bruker D8 Advance with Cu $K\alpha_1$ radiation at room temperature and elemental analysis (EA). IR spectra of the olsalazine sodium and [Mg_2(olsalazine)$(DMF)_2$]·2DMF·3H_2O were recorded on a Perkin Elmer instrument Spectrum 100. Thermogravimetric analysis of [Mg_2(olsalazine)$(DMF)_2$]·2DMF·3H_2O was performed under N_2 atmosphere using a TGA@500-2338 instrument. The MOF was heated with a rate of 1 ^oC/min up to 850 ^oC.

S3. Additional characterization of Mg_2(olsalazine)

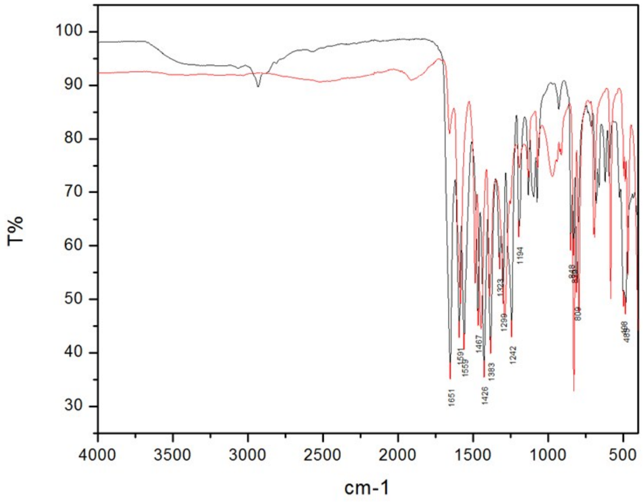

Figure S2. IR spectra of free olsalazine (red) and [Mg_2(olsalazine)$(DMF)_2$]·guest molecules (black).

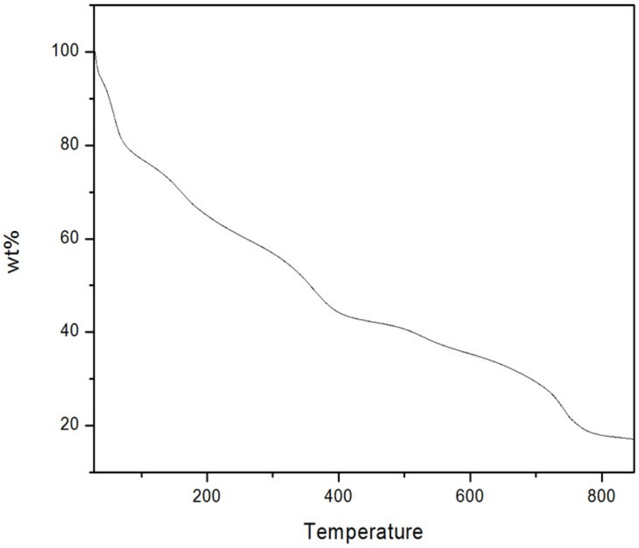

Figure S3. Thermogravimetric analysis of [Mg_2(olsalazine)$(DMF)_2$]·2DMF·3H_2O shows a weight loss of 46.8 % at 370 ^oC corresponding to the loss of coordinated to Mg(II) and guest DMF and H_2O molecules (48.9 %). The framework is stable up to 500 ^oC.

S4. MIL-53 constraints example

To graphically demonstrate that the building algorithm can be applied to 1-D rod MOFs other than MOF-74 in the **etb** net, we recreate Figure 5 from the article but for a different 1-D rod MOF, namely MIL-53.[3] The same constraints would exist for enumerating MIL-53 analogs and the same design variables (F, dC) would need to be solved. The (F, dC) combination leading to the smallest RMSD fit between the proposed ligand and the new rod locations would be identified as the best crystal structure.

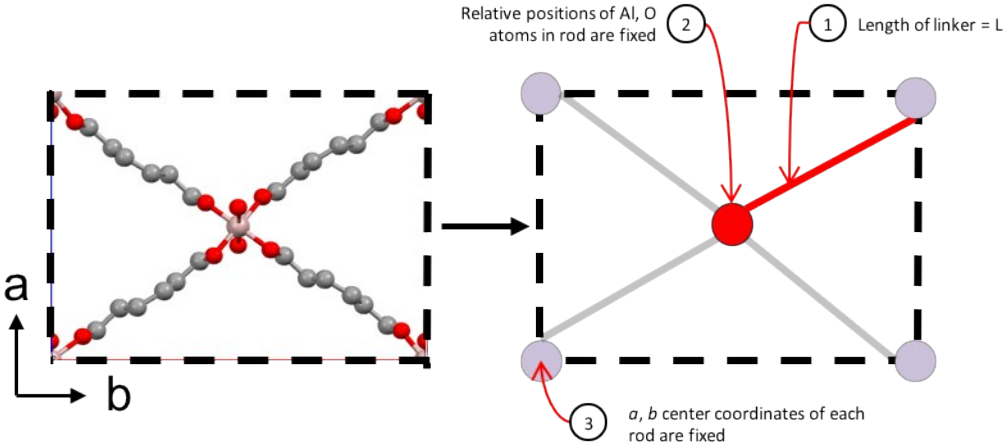

Figure S4. The simplification of MIL-53 to a set of geometric constraints and design variables shows how the building algorithm can be applied to MOFs other than MOF-74 in the **etb** net. In fact, this example demonstrates how no information about the underlying net of MIL-53 is necessary to set up a geometric optimization to enumerate analogs of the original structure.

S5. CIF files for MOF-74 analog library

Unzip the <analogs.zip> zip file for structures and charges. The <.cssr> file provides the crystallographic structure with each atom's unique type and the <.def> file includes the charges for each unique atom type in the structure. The un-typed structures are also provided in the bare <.cif> files.

S6. Experimental adsorption studies

Experimental procedure

All sorption isotherms (N_2 was collected at 77 K and 1 bar whereas CO_2 adsorption isotherms were collected at 298, 303 and 313 K and 1 bar) were measured on [Mg_2(olsalazine)] using the Intelligent Gravimetric Analyser (IGA) from Hiden. The IGA is a fully computer controlled microbalance in an ultra-high vacuum (UHV) system and has both pressure and temperature regulation systems. The microbalance has a long-term stability of 1 mg with a weighing resolution of 0.2 mg. Adsorption CO_2 isotherms at 298, 303 and 313 K and 1 bar were obtained using a circulating water bath to control the sample temperature (with accuracy of ± 0.1 ^oC). The pressure and temperature were both controlled by computer using IGA software during isotherm measurements.

Activation

Mg_2(olsalazine)($DMF)_2$ was initially immersed in liquid methanol overnight, filtered and 27.1438 mg of sample was loaded within IGA and heated at 393 K under high vacuum (10^{-8} mbar) for 12 hours until a plateau was observed (weight loss of 37%). When the material was further heated at 423-453 K, we have observed no additional weight loss confirming that the material was successfully activated at 293 K and that the measurements were performed on [Mg_2(olsalazine)].

CO_2 Isotherms and isosteric adsorption heat

The CO_2 adsorption isotherms were analyzed utilizing the virial-type expression, using the adsorption branches measured at 298, 303 and 313 K (1):

$$\ln(N/P) = A_0 + A_1N + A_2N^2 + \ldots \qquad (1)$$

where: P is the pressure (Pa), N is the amount adsorbed (mol/g), A_n are the virial coefficients. At low uptakes, a plot on ln(N/P) versus N is linear with gradient A_1 and intercept A_0. A_0 is exp(K_H), where K_H is the Henry's law constant. The isosteric adsorption heat at zero coverage Qst_0 is expressed as (2):

$$Qst_0 = R\frac{\partial A_0}{\partial(1/T)} \qquad (2)$$

where: R is gas constant (8.314 J K^-1 mol^{-1}) and T is the adsorption temperature (K). A plot of A_0 versus 1/T has a gradient of Qst_0/R and this was used to calculate Qst_0. The Qst_0 is calculated to be 36.1 kJ/mol, which is in close agreement with the theoretical value of 39.0 kJ/mol computed from GCMC calculations.

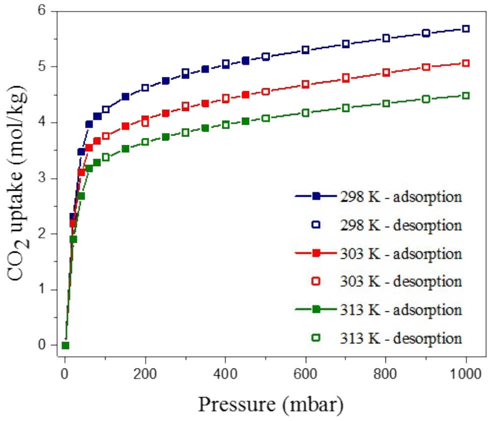

Figure S5: Experimental CO_2 isotherms measured on Mg_2(olsalazine) - filled squares: adsorption, empty squares: desorption.

N_2 Isotherm

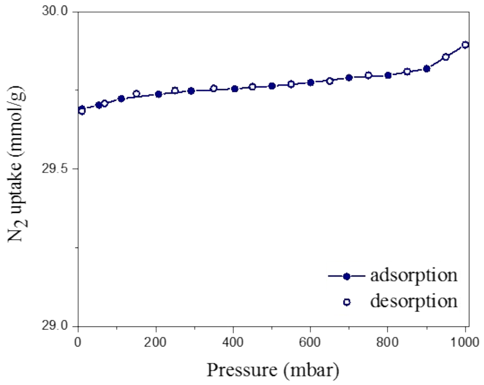

Figure S6: N_2 isotherm measured at 77K and 1 bar on Mg_2(olsalazine). The Brunauer–Emmett–Teller (BET) model applied over p/p^0 = 0.02–0.13 gives a surface area of 2331.8 m^2/g.

S7. ESI References

1 L. C. Lin, K. Lee, L. Gagliardi, J. B. Neaton and B. Smit, *J. Chem. Theory Comput.*, 2014, **10**, 1477–1488.

2 J. a. Mason, K. Sumida, Z. R. Herm, R. Krishna and J. R. Long, *Energy Environ. Sci.*, 2011, **4**, 3030.

3 T. Loiseau, C. Serre, C. Huguenard, G. Fink, F. Taulelle, M. Henry, T. Bataille and G. Férey, *Chem. Eur. J.*, 2004, **10**, 1373–1382.
